# (3*R*,4*R*)-2,5-Dioxo-1-*m*-tolyl-3,4-diyl diacetate

**DOI:** 10.1107/S1600536809020637

**Published:** 2009-06-06

**Authors:** Sara Naz, Javid Zaidi, Tahir Mehmood, Peter G. Jones

**Affiliations:** a Department of Chemistry, Quaid-I-Azam University, Islamabad 45320, Pakistan; b Institut for Anorganische und Analytische Chemie, Technische Universität Braunschweig, Hagenring 30, 38106 Braunschweig, Germany

## Abstract

In the enanti­omerically pure title compound, C_15_H_15_NO_6_, the five-membered ring displays a twist conformation with the local axis through the N atom. The acetyl groups are perpendicular to the ring [dihedral angles 80.3 (1) and 89.3 (1)°] and project to opposite sides. The packing is governed by two weak C—H⋯O inter­actions, forming layers of mol­ecules parallel to the *ab* plane.

## Related literature

For the potential biological activity, pharmaceutical utility and biological effects of cyclic imides, see: Adomat & Böger (2000 ▶); Böger & Wakabayashi (1995 ▶); Birchfield & Casida (1997 ▶); Cechinel Filho, Nunes, Calixto & Yunes (1995 ▶); Cechinel Filho, de Campos, Corrêa, Yunes & Nunes (2003 ▶); López *et al.* (2003 ▶); Lima *et al.* (1999 ▶); Sami *et al.* (2000 ▶); Wang *et al.* (2000 ▶); Watanabe *et al.* (1998 ▶).
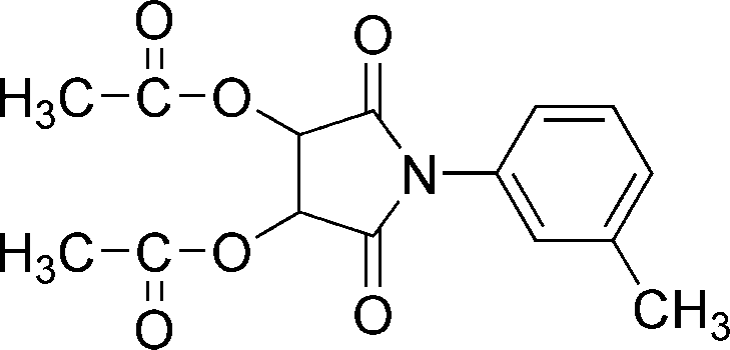


## Experimental

### 

#### Crystal data

C_15_H_15_NO_6_
*M* *_r_* = 305.28
Monoclinic, 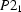

*a* = 8.2382 (4) Å
*b* = 5.5380 (3) Å
*c* = 16.6015 (9) Å
β = 103.664 (5)°
*V* = 735.98 (7) Å^3^
*Z* = 2
Cu *K*α radiation
μ = 0.91 mm^−1^
*T* = 100 K
0.20 × 0.15 × 0.08 mm

#### Data collection

Oxford Diffraction Nova A diffractometer
Absorption correction: multi-scan (CrysAlisPro; Oxford Diffraction, 2008 ▶) *T*_min_ = 0.892, *T*_max_ = 1.000 (expected range = 0.829–0.930)
15796 measured reflections
2790 independent reflections
2742 reflections with *I* > 2σ(*I*)
*R*_int_ = 0.030

#### Refinement

*R*[*F* ^2^ > 2σ(*F* ^2^)] = 0.026
*wR*(*F* ^2^) = 0.067
*S* = 1.04
2790 reflections
202 parameters
1 restraint
H-atom parameters constrained
Δρ_max_ = 0.15 e Å^−3^
Δρ_min_ = −0.18 e Å^−3^
Absolute structure: Flack (1983 ▶), 1097 Friedel pairs
Flack parameter: 0.04 (12)

### 

Data collection: *CrysAlisPro* (Oxford Diffraction, 2008 ▶); cell refinement: *CrysAlisPro*; data reduction: *CrysAlisPro*; program(s) used to solve structure: *SHELXS97* (Sheldrick, 2008 ▶); program(s) used to refine structure: *SHELXL97* (Sheldrick, 2008 ▶); molecular graphics: *XP* (Siemens, 1994 ▶); software used to prepare material for publication: *SHELXL97*.

## Supplementary Material

Crystal structure: contains datablocks I, global. DOI: 10.1107/S1600536809020637/at2801sup1.cif

Structure factors: contains datablocks I. DOI: 10.1107/S1600536809020637/at2801Isup2.hkl

Additional supplementary materials:  crystallographic information; 3D view; checkCIF report

## Figures and Tables

**Table 1:** Hydrogen-bond geometry (Å, °)

*D*—H⋯*A*	*D*—H	H⋯*A*	*D*⋯*A*	*D*—H⋯*A*
C12—H12*C*⋯O1^i^	0.98	2.25	3.1957 (17)	161
C15—H15*A*⋯O2^ii^	0.98	2.52	3.4104 (17)	150

Symmetry codes: (i) 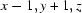; (ii) 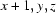.

**Fig. 1.**
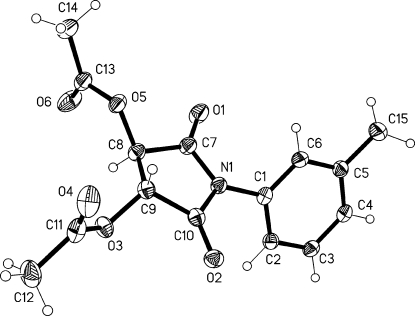
The molecule of the title compound in the crystal. Ellipsoids correspond to 50% probability levels.

**Fig. 2.**
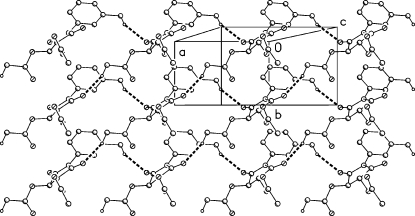
Packing diagram of the title compound viewed perpendicular to the ab plane. H bonds are indicated as thick dashed lines. H atoms not involved in H bonds are omitted.

## supplementary crystallographic information

### Comment

Synthetic cyclic imides, such as succinimides, maleimides, glutarimides, phthalimides and related compounds, possess structural features that confer potential biological activity and pharmaceutical utility. All these classes of cyclic imides have received attention because of their antibacterial, antifungal, analgesic (Cechinel *et al.*, 2003) and antitumor activities (Sami *et al.*, 2000; Wang *et al.*, 2000). Some of these effects appear to be related to the size and electrophilic characteristics of substituent groups on the imide ring, which can modify its steric properties (Cechinel *et al.*, 1995; Lima *et al.*, 1999; López *et al.*, 2003). Beside these interesting biological effects, some cyclic imides, *e.g.*, chlorophthalim (Adomat & Böger, 2000), *N*-aryltetrahydrophthalimide (Birchfield & Casida, 1997) and N– (4-chloro-2-fluoro-5-propargyloxy)-phenyl-3,4,5,6-tetrahydrophthalimide (Watanabe *et al.*, 1998) are peroxidizing herbicides, a class of herbicides that inhibit protoporphyrinogen IX oxidase, a key enzyme of heme and chlorophyll biosynthesis (Böger & Wakabayashi, 1995). In the course of our studies of cyclic imides, we have synthesized the title compound (I) and here present its structure (Fig. 1).

In the title molecule (I), the five-membered ring displays a twist conformation with the local twofold axis through N1 and the midpoint of C8—C9. The toluene ring subtends an interplanar angle of 51.3 (1)° with the planar moiety N1/C7/C10/O1/O2. The acetyl groups are perpendicular to the pyrrolidine moiety, whereby the plane C7–C9 subtends an angle of 80.3 (1)° to the plane C8/O5/O6/ C13/C14 and C8–C10 an angle of 89.3 (1)° to C9/O3/O4/C11/C12; the carbonyl O atoms project to opposite sides of the ring. The packing (Fig. 2) consists of broad layers of molecules parallel to the *ab* plane at *z*≈ 1/4, 3/4, the molecules being linked by the weak interactions H15A···O2 [2.52 Å] and H12C···O1 [2.25 Å].

### Experimental

The title compound was synthesized from 0.005 mole (1.08 g) diacetyl-*L*-tartaric acid anhydride (2,5-dioxotetrahydrofuran-3,4-diyldiacetate), 3-methylaniline (0.005 mole, 0.53 g) and 10 ml of glacial acetic acid. The mixture was refluxed for 1 h under nitrogen. Glacial acetic acid was removed by extracting the reaction mixture with ethyl acetate and water. The product was purified by column chromatography and recrystallized from dry ethanol [yield 68%; m.p. 379–381 K].

### Refinement

Methyl H atoms were located in difference syntheses, idealized to C—H 0.98 Å and H—C—H 109.5°, and refined as rigid groups allowed to rotate but not tip. Other H atoms were placed in calculated positions and refined using a riding model with C—H 0.95 Å for aromatic H and 1.00 Å for methine CH. Hydrogen *U* values were fixed at 1.5 × *U*(eq) of the parent atom for methyl H and 1.2 × *U*(eq) of the parent atom for other H. The compound is enantiomerically pure and its absolute configuration (*R* at C8 and C9, crystallographic numbering) was confirmed by the Flack (1983) parameter. Data are 99.7% complete to 2θ 145°.

### Crystal data

**Table:**

C_15_H_15_NO_6_	*F*(000) = 320
*M**_r_* = 305.28	*D*_x_ = 1.378 Mg m^−^^3^
Monoclinic, *P*2_1_	Cu *K*α radiation, λ = 1.54184 Å
Hall symbol: P 2yb	Cell parameters from 13866 reflections
*a* = 8.2382 (4) Å	θ = 5.5–75.8°
*b* = 5.5380 (3) Å	µ = 0.91 mm^−^^1^
*c* = 16.6015 (9) Å	*T* = 100 K
β = 103.664 (5)°	Tablet, colourless
*V* = 735.98 (7) Å^3^	0.20 × 0.15 × 0.08 mm
*Z* = 2	

### Data collection

**Table:**

Oxford Diffraction Nova A diffractometer	2790 independent reflections
Radiation source: Nova (Cu) X-ray Source	2742 reflections with *I* > 2σ(*I*)
mirror	*R*_int_ = 0.030
Detector resolution: 10.3543 pixels mm^-1^	θ_max_ = 75.9°, θ_min_ = 5.5°
ω–scan	*h* = −10→10
Absorption correction: multi-scan (*CrysAlis PRO*; Oxford Diffraction, 2008)	*k* = −6→6
*T*_min_ = 0.892, *T*_max_ = 1.000	*l* = −20→20
15796 measured reflections	

### Refinement

**Table:**

Refinement on *F*^2^	Secondary atom site location: difference Fourier map
Least-squares matrix: full	Hydrogen site location: inferred from neighbouring sites
*R*[*F*^2^ > 2σ(*F*^2^)] = 0.026	H-atom parameters constrained
*wR*(*F*^2^) = 0.067	*w* = 1/[σ^2^(*F*_o_^2^) + (0.041*P*)^2^ + 0.1139*P*] where *P* = (*F*_o_^2^ + 2*F*_c_^2^)/3
*S* = 1.04	(Δ/σ)_max_ = 0.001
2790 reflections	Δρ_max_ = 0.14 e Å^−^^3^
202 parameters	Δρ_min_ = −0.18 e Å^−^^3^
1 restraint	Absolute structure: Flack (1983), 1097 Freidel pairs
Primary atom site location: structure-invariant direct methods	Flack parameter: 0.04 (12)

### Special details

**Table:**

Geometry. All e.s.d.'s (except the e.s.d. in the dihedral angle between two l.s. planes) are estimated using the full covariance matrix. The cell e.s.d.'s are taken into account individually in the estimation of e.s.d.'s in distances, angles and torsion angles; correlations between e.s.d.'s in cell parameters are only used when they are defined by crystal symmetry. An approximate (isotropic) treatment of cell e.s.d.'s is used for estimating e.s.d.'s involving l.s. planes.
Refinement. Refinement of *F*^2^ against ALL reflections. The weighted *R*-factor *wR* and goodness of fit *S* are based on *F*^2^, conventional *R*-factors *R* are based on *F*, with *F* set to zero for negative *F*^2^. The threshold expression of *F*^2^ > σ(*F*^2^) is used only for calculating *R*-factors(gt) *etc*. and is not relevant to the choice of reflections for refinement. *R*-factors based on *F*^2^ are statistically about twice as large as those based on *F*, and *R*- factors based on ALL data will be even larger.

### Fractional atomic coordinates and isotropic or equivalent isotropic displacement parameters (Å^2^)

**Table:**

	*x*	*y*	*z*	*U*_iso_*/*U*_eq_	
N1	0.83245 (11)	0.4087 (2)	0.77707 (5)	0.0201 (2)	
O1	0.95264 (12)	0.3617 (2)	0.66503 (5)	0.0332 (2)	
O2	0.65363 (10)	0.53516 (17)	0.85796 (5)	0.02392 (19)	
O3	0.45314 (10)	0.70803 (16)	0.70307 (5)	0.02366 (19)	
O4	0.46297 (13)	1.11400 (18)	0.71612 (6)	0.0353 (2)	
O5	0.73911 (11)	0.75624 (17)	0.59745 (5)	0.02408 (19)	
O6	0.63863 (14)	0.5220 (2)	0.48590 (6)	0.0378 (2)	
C1	0.93789 (14)	0.2475 (2)	0.83483 (7)	0.0201 (2)	
C2	0.86415 (14)	0.0719 (2)	0.87400 (7)	0.0211 (2)	
H2	0.7461	0.0634	0.8653	0.025*	
C3	0.96605 (14)	−0.0911 (3)	0.92619 (7)	0.0225 (2)	
H3	0.9175	−0.2128	0.9534	0.027*	
C4	1.13842 (14)	−0.0773 (3)	0.93890 (7)	0.0228 (2)	
H4	1.2066	−0.1920	0.9739	0.027*	
C5	1.21311 (14)	0.1025 (2)	0.90112 (7)	0.0222 (2)	
C6	1.11073 (15)	0.2667 (2)	0.84868 (7)	0.0221 (2)	
H6	1.1589	0.3912	0.8225	0.026*	
C7	0.84338 (14)	0.4369 (3)	0.69489 (7)	0.0232 (2)	
C8	0.69155 (14)	0.5797 (2)	0.64972 (7)	0.0215 (2)	
H8	0.6045	0.4694	0.6169	0.026*	
C9	0.62962 (14)	0.6982 (2)	0.71900 (7)	0.0211 (2)	
H9	0.6769	0.8647	0.7290	0.025*	
C10	0.69933 (14)	0.5396 (2)	0.79435 (7)	0.0203 (2)	
C11	0.38285 (15)	0.9315 (3)	0.70241 (7)	0.0259 (3)	
C12	0.19739 (16)	0.9069 (3)	0.68349 (9)	0.0356 (3)	
H12A	0.1653	0.8170	0.7282	0.053*	
H12B	0.1588	0.8199	0.6311	0.053*	
H12C	0.1463	1.0676	0.6788	0.053*	
C13	0.70695 (15)	0.7034 (3)	0.51479 (7)	0.0252 (3)	
C14	0.77036 (16)	0.9016 (3)	0.46948 (8)	0.0314 (3)	
H14A	0.8918	0.8869	0.4779	0.047*	
H14B	0.7432	1.0580	0.4906	0.047*	
H14C	0.7177	0.8899	0.4102	0.047*	
C15	1.40119 (15)	0.1163 (3)	0.91705 (8)	0.0317 (3)	
H15A	1.4327	0.2568	0.8882	0.048*	
H15B	1.4438	−0.0310	0.8966	0.048*	
H15C	1.4492	0.1322	0.9767	0.048*	

### Atomic displacement parameters (Å^2^)

**Table:**

	*U*^11^	*U*^22^	*U*^33^	*U*^12^	*U*^13^	*U*^23^
N1	0.0208 (4)	0.0202 (5)	0.0192 (4)	0.0022 (4)	0.0047 (3)	0.0014 (4)
O1	0.0348 (5)	0.0416 (6)	0.0264 (4)	0.0167 (4)	0.0137 (4)	0.0088 (4)
O2	0.0247 (4)	0.0258 (5)	0.0224 (4)	−0.0010 (3)	0.0078 (3)	−0.0023 (4)
O3	0.0208 (4)	0.0199 (5)	0.0293 (4)	0.0049 (3)	0.0040 (3)	−0.0012 (4)
O4	0.0435 (5)	0.0218 (5)	0.0354 (5)	0.0086 (4)	−0.0010 (4)	−0.0048 (4)
O5	0.0279 (4)	0.0234 (5)	0.0201 (4)	−0.0003 (4)	0.0041 (3)	0.0043 (3)
O6	0.0587 (6)	0.0312 (5)	0.0242 (4)	−0.0094 (5)	0.0113 (4)	−0.0017 (4)
C1	0.0242 (5)	0.0179 (6)	0.0175 (5)	0.0023 (5)	0.0033 (4)	−0.0004 (5)
C2	0.0218 (5)	0.0217 (7)	0.0197 (5)	−0.0014 (5)	0.0045 (4)	−0.0015 (5)
C3	0.0277 (5)	0.0204 (6)	0.0195 (5)	−0.0025 (5)	0.0058 (4)	0.0008 (5)
C4	0.0270 (5)	0.0227 (6)	0.0180 (5)	0.0039 (5)	0.0038 (4)	0.0016 (5)
C5	0.0221 (5)	0.0254 (6)	0.0190 (5)	0.0000 (5)	0.0044 (4)	−0.0019 (5)
C6	0.0235 (5)	0.0225 (6)	0.0208 (5)	−0.0005 (5)	0.0066 (4)	0.0025 (5)
C7	0.0253 (5)	0.0228 (6)	0.0219 (5)	0.0020 (5)	0.0063 (4)	0.0028 (5)
C8	0.0243 (5)	0.0192 (6)	0.0211 (5)	0.0019 (5)	0.0058 (4)	0.0019 (5)
C9	0.0209 (5)	0.0191 (6)	0.0225 (5)	0.0014 (5)	0.0040 (4)	−0.0003 (5)
C10	0.0198 (5)	0.0178 (6)	0.0225 (5)	−0.0013 (4)	0.0034 (4)	−0.0017 (5)
C11	0.0337 (6)	0.0238 (7)	0.0186 (5)	0.0096 (6)	0.0030 (4)	−0.0025 (5)
C12	0.0298 (6)	0.0397 (9)	0.0357 (6)	0.0147 (7)	0.0046 (5)	−0.0028 (7)
C13	0.0285 (6)	0.0256 (7)	0.0219 (5)	0.0036 (5)	0.0065 (4)	0.0043 (5)
C14	0.0374 (6)	0.0307 (8)	0.0271 (6)	0.0003 (6)	0.0096 (5)	0.0077 (6)
C15	0.0233 (6)	0.0380 (9)	0.0333 (7)	0.0007 (5)	0.0056 (5)	0.0062 (6)

### Geometric parameters (Å, °)

**Table:**

N1—C7	1.3971 (14)	C8—C9	1.5136 (16)
N1—C10	1.3996 (15)	C9—C10	1.5252 (17)
N1—C1	1.4413 (15)	C11—C12	1.4916 (17)
O1—C7	1.2000 (15)	C13—C14	1.4931 (18)
O2—C10	1.2023 (14)	C2—H2	0.9500
O3—C11	1.3654 (16)	C3—H3	0.9500
O3—C9	1.4160 (13)	C4—H4	0.9500
O4—C11	1.1990 (18)	C6—H6	0.9500
O5—C13	1.3664 (15)	C8—H8	1.0000
O5—C8	1.4225 (15)	C9—H9	1.0000
O6—C13	1.1952 (18)	C12—H12A	0.9800
C1—C2	1.3873 (17)	C12—H12B	0.9800
C1—C6	1.3918 (16)	C12—H12C	0.9800
C2—C3	1.3881 (17)	C14—H14A	0.9800
C3—C4	1.3876 (16)	C14—H14B	0.9800
C4—C5	1.3949 (18)	C14—H14C	0.9800
C5—C6	1.3962 (17)	C15—H15A	0.9800
C5—C15	1.5110 (15)	C15—H15B	0.9800
C7—C8	1.5196 (16)	C15—H15C	0.9800
			
C7—N1—C10	112.13 (10)	C1—C2—H2	120.6
C7—N1—C1	123.44 (10)	C3—C2—H2	120.6
C10—N1—C1	124.24 (9)	C4—C3—H3	119.8
C11—O3—C9	116.83 (10)	C2—C3—H3	119.8
C13—O5—C8	116.70 (10)	C3—C4—H4	119.5
C2—C1—C6	121.35 (11)	C5—C4—H4	119.5
C2—C1—N1	118.97 (10)	C1—C6—H6	120.1
C6—C1—N1	119.66 (11)	C5—C6—H6	120.1
C1—C2—C3	118.78 (11)	O5—C8—H8	110.6
C4—C3—C2	120.35 (12)	C9—C8—H8	110.6
C3—C4—C5	121.02 (11)	C7—C8—H8	110.6
C4—C5—C6	118.67 (10)	O3—C9—H9	109.8
C4—C5—C15	120.03 (11)	C8—C9—H9	109.8
C6—C5—C15	121.31 (11)	C10—C9—H9	109.8
C1—C6—C5	119.80 (11)	C11—C12—H12A	109.5
O1—C7—N1	126.33 (11)	C11—C12—H12B	109.5
O1—C7—C8	125.94 (10)	H12A—C12—H12B	109.5
N1—C7—C8	107.73 (9)	C11—C12—H12C	109.5
O5—C8—C9	110.88 (10)	H12A—C12—H12C	109.5
O5—C8—C7	110.22 (9)	H12B—C12—H12C	109.5
C9—C8—C7	103.69 (9)	C13—C14—H14A	109.5
O3—C9—C8	112.97 (9)	C13—C14—H14B	109.5
O3—C9—C10	110.28 (9)	H14A—C14—H14B	109.5
C8—C9—C10	104.14 (10)	C13—C14—H14C	109.5
O2—C10—N1	126.34 (11)	H14A—C14—H14C	109.5
O2—C10—C9	126.64 (11)	H14B—C14—H14C	109.5
N1—C10—C9	106.95 (9)	C5—C15—H15A	109.5
O4—C11—O3	123.30 (11)	C5—C15—H15B	109.5
O4—C11—C12	127.42 (13)	H15A—C15—H15B	109.5
O3—C11—C12	109.28 (12)	C5—C15—H15C	109.5
O6—C13—O5	122.99 (12)	H15A—C15—H15C	109.5
O6—C13—C14	127.10 (12)	H15B—C15—H15C	109.5
O5—C13—C14	109.91 (11)		
			
C7—N1—C1—C2	−123.73 (13)	N1—C7—C8—O5	137.39 (10)
C10—N1—C1—C2	50.86 (16)	O1—C7—C8—C9	−161.45 (14)
C7—N1—C1—C6	54.68 (16)	N1—C7—C8—C9	18.66 (14)
C10—N1—C1—C6	−130.73 (12)	C11—O3—C9—C8	−121.50 (11)
C6—C1—C2—C3	−1.77 (18)	C11—O3—C9—C10	122.43 (11)
N1—C1—C2—C3	176.61 (10)	O5—C8—C9—O3	99.30 (11)
C1—C2—C3—C4	0.19 (18)	C7—C8—C9—O3	−142.43 (10)
C2—C3—C4—C5	1.31 (18)	O5—C8—C9—C10	−141.03 (9)
C3—C4—C5—C6	−1.23 (17)	C7—C8—C9—C10	−22.76 (12)
C3—C4—C5—C15	178.99 (12)	C7—N1—C10—O2	174.41 (12)
C2—C1—C6—C5	1.85 (18)	C1—N1—C10—O2	−0.7 (2)
N1—C1—C6—C5	−176.52 (11)	C7—N1—C10—C9	−8.51 (14)
C4—C5—C6—C1	−0.33 (17)	C1—N1—C10—C9	176.36 (11)
C15—C5—C6—C1	179.45 (12)	O3—C9—C10—O2	−41.62 (17)
C10—N1—C7—O1	173.58 (14)	C8—C9—C10—O2	−163.10 (12)
C1—N1—C7—O1	−11.3 (2)	O3—C9—C10—N1	141.31 (10)
C10—N1—C7—C8	−6.52 (15)	C8—C9—C10—N1	19.83 (12)
C1—N1—C7—C8	168.64 (11)	C9—O3—C11—O4	−1.53 (16)
C13—O5—C8—C9	−144.04 (10)	C9—O3—C11—C12	179.15 (9)
C13—O5—C8—C7	101.73 (12)	C8—O5—C13—O6	1.85 (18)
O1—C7—C8—O5	−42.72 (18)	C8—O5—C13—C14	−177.72 (10)

### Hydrogen-bond geometry (Å, °)

**Table:**

*D*—H···*A*	*D*—H	H···*A*	*D*···*A*	*D*—H···*A*
C12—H12C···O1^i^	0.98	2.25	3.1957 (17)	161
C15—H15A···O2^ii^	0.98	2.52	3.4104 (17)	150

Symmetry codes: (i) *x*−1, *y*+1, *z*; (ii) *x*+1, *y*, *z*.
